# Joint association of sleep quality and physical activity with metabolic dysfunction-associated fatty liver disease: a population-based cross-sectional study in Western China

**DOI:** 10.1038/s41387-024-00312-3

**Published:** 2024-07-22

**Authors:** Ying Wang, Qian Zhao, Jialu Yang, Yushan Wang, Lei Deng, Hamulati Xieyire, Tuerxun Gulijiehere, Mutalifu Munire, Fen Liu, Xiaomei Li, Min Xia, Yan Liu, Yining Yang

**Affiliations:** 1 State Key Laboratory of Pathogenesis, Prevention and Treatment of High Incidence Diseases in Central Asia, Department of Cardiology, First Affiliated Hospital of Xinjiang Medical University, Urumqi, China; 2 Xinjiang Key Laboratory of Cardiovascular Disease Research, Clinical Medical Research Institute of Xinjiang Medical University, Urumqi, China; 3 Guangdong Provincial Key Laboratory of Food, Nutrition and Health, and Department of Nutrition, School of Public Health, Sun Yat-sen University, Guangzhou, China; 4 Center of Health Management, The First Affiliated Hospital of Xinjiang Medical University, Urumqi, China; 5 Baoshihua Korla Hospital, Korla, China; 6 Department of Cardiology, People’s Hospital of Xinjiang Uygur Autonomous Region, Urumqi, China

**Keywords:** Endocrine system and metabolic diseases, Risk factors, Epidemiology

## Abstract

**Background:**

Metabolic dysfunction-associated fatty liver disease (MAFLD) is a growing threat leading to substantial disease burden globally. Poor sleep and physical inactivity are common in modern societies and independently associated with MAFLD, however, their joint effects on MAFLD remains unclear.

**Methods:**

This population-based cross-sectional study was conducted in Xinjiang Uygur Autonomous Region, China, between July 2019 and September 2021. Self-reported sleep behaviors and physical activity (PA) were assessed using validated questionnaires. The primary outcome was radiological diagnosis of MAFLD.

**Results:**

Of the 10 089 participants aged 47.0 (9.1) years (51.6% men), 3854 (38.2%) individuals had MAFLD. Poor sleep quality and physical inactivity were independently and jointly associated with an increased prevalence of MAFLD, independent of traditional risk factors (*P* < 0.05). Compared to subjects with guideline-recommended moderate-to-vigorous PA (MVPA) and good sleep quality, individuals with no recommended MVPA and poor sleep had the highest possibility of MAFLD (odds ratio = 2.36, 95% confidence interval: 1.81 – 3.08). Enhancing sleep quality substantially attenuated MAFLD prevalence regardless of the volume of PA, whereas, engaging in PA well above current guidelines did not adequately counteract the adverse impacts of poor sleep on MAFLD.

**Conclusions:**

Public health awareness and strategies concurrently targeting both sleep quality and PA should be encouraged to curb the climbing prevalence of MAFLD.

## Introduction

Metabolic dysfunction-associated fatty liver disease (MAFLD) has emerged as a substantial public health concern, impacting nearly 25% of the global adult population [1]. Concurrently with an increase in unhealthy lifestyles, the prevalence of MAFLD has risen from 22.8% to 35.6% in China between 2009 and 2017 [2]. MAFLD may not only progress to end-stage liver diseases, but also lead to various extrahepatic complications [3–5], resulting in a heavy burden to all societies.

Physical inactivity and poor sleep quality, two major lifestyle behaviors increasingly prevalent in modern societies, have been found to be adversely associated with various metabolic dysfunctions [6–13]. Adherence to sufficient physical activity (PA) is essential for ameliorating insulin resistance and maintaining optimal body weight, the cornerstone for the management of MAFLD [6, 7]. On the other hand, various characteristics of sleep quality, such as short sleep duration [8, 9], insomnia [10, 11] and habitual snoring [12, 13], are reported to be positively associated with metabolic disorders. Notably, both PA and sleep quality are complex, co-dependent and may influence metabolic homeostasis through related pathways [14–16]. For example, PA was reported to exert a short-term effect on sleep duration [15] and help maintain a stable circadian rhythm [16]. Due to the temporal dependence between these two behaviors, extended sleep duration could consequently lead to a decrease in the duration of PA [14, 17]. Although the independent associations between PA, sleep quality and MAFLD have been found in several cohorts [7, 13, 18, 19], to the best of our knowledge, there is limited study interrogating the potential joint effect of PA and sleep quality on MAFLD.

In our study, the objective was to investigate the combined impact of PA and a comprehensive evaluation of sleep quality (including daytime napping, which is uncommon in Western societies but prevalent in China [20]) on the possibility of MAFLD in Western China.

## Methods

### Study population

The data utilized in this research was sourced from the baseline survey of the Population-based Cohort study of Chronic Diseases in Xinjiang (PCCDX), which was carried out from July 2019 to September 2021. The PCCDX was a community-based prospective cohort study conducted in Urumqi and Korla, using a two-stage stratified strategy. During the period spanning from July 2019 to September 2021, a cohort of 12 295 individuals between the ages of 30 and 74, who did not exhibit severe disabilities, were enrolled in the initial survey and subsequently underwent liver ultrasound examinations. The research received ethical approval from the Ethics Committee of the First Affiliated Hospital of Xinjiang Medical University (K201705-02, K202101-20) and adhered to the principles outlined in the Declaration of Helsinki. All participants provided written informed consent before participating in the study. Detailed information on the physical examination, laboratory measurements, and demographic data collection in the PCCDX were provided in the Supplementary Methods.

In this study, 2206 participants were excluded for the following reasons: (1) insufficient data for diagnosing MAFLD (*n* = 979); (2) history of liver cirrhosis, liver resection, or liver cancer (*n* = 13); (3) extreme outliers for waist circumference and body mass index (BMI) which exceeded 3 standard deviations (*n* = 582); and (4) missing values for sleep behaviors (*n* = 632) (Table S1 and Figure S1). The definition and detailed assessment of MAFLD were provided in the Supplementary Methods.

### Assessment of sleep quality and PA

Self-reported sleep behaviors were assessed with the Pittsburgh Sleep Quality Index questionnaire [21]. Given a 2 h jet lag between Beijing and Xinjiang Uygur Autonomous Region, bedtime was categorized into 3 groups according to Beijing Time: before 0:00 am, between 0:00 and 1:00 am, and after 1:00 am. Sleep duration during the night was categorized as follows: short sleep (< 7 h/day), normal sleep (7–8 h/day), and long sleep (> 8 h/day). Insomnia, snoring, and excessive daytime sleepiness were classified based on the frequency of occurrence: never/rarely (less than one episode per week), sometimes (one or two episodes per week), or usually (three or more episodes per week). Daytime napping was divided into two categories: 0–30 min per day and more than 30 min per day. Due to the highly interconnected nature of sleep behaviors and their potential synergistic effects on metabolic homeostasis, a composite score was calculated from the six dimensions to obtain a comprehensive evaluation of overall sleep quality. The healthy sleep score, as assessed on a scale from 0 to 6, demonstrated a positive correlation with sleep quality, with higher scores reflecting superior sleep patterns [13]. Following the results of the restricted cubic spline analysis, overall sleep quality was classified into three distinct categories: good (healthy sleep score of 5 or higher), intermediate (healthy sleep score ranging from 3 to 4), and poor (healthy sleep score of 2 or lower).

The International Physical Activity Questionnaire (IPAQ) was employed to assess and quantify PA [22]. Total weekly metabolic equivalent (MET) was used as a measurement of PA, derived by multiplying the MET values corresponding to activities by the weekly duration of PA in hours. As per the findings of the PURE study [23], PA was further categorized as low (< 600 MET × minutes per week), moderate (600–3000 MET × minutes per week), and high (> 3000 MET × minutes per week). Additionally, according to the recommendation (≥ 150 min moderate PA, or ≥ 75 min vigorous PA, or equivalent combinations of both throughout the week) proposed by the World Health Organization (WHO) [24], moderate-to-vigorous PA (MVPA) was further divided into two groups (meeting, or not meeting the recommended target).

### Statistical analysis

The study participants’ basic characteristics were summarized using mean (SD) or median (interquartile range) for continuous variables and n (%) for categorical variables, stratified by the presence of MAFLD. Trend analysis for continuous variables was conducted using a linear regression model, while the Mantel-Haenszel chi-squared test was employed for categorical variables. Missing values were imputed using the Multiple Imputation by Chained Equations (MICE) method [25] as described in Table S2.

The minimum sufficiently adjusted set [26, 27] was selected using a directed acyclic graph (Fig. S2). Independent associations of PA and sleep quality as categorical variables with MAFLD were examined by multivariable logistic regression analyses, adjusting for age, gender, higher education (yes or no), married (yes or no), current smoking (yes or no), alcohol drinking (yes or no), sedentary time, diet diversity score and BMI status and sleep quality/PA when appropriate. Additionally, as continuous variables, the potential nonlinear relationship of PA and sleep quality with MAFLD were estimated by a restricted cubic spline fitted in the fully adjusted logistic regression model.

Multivariable logistic regression models were employed to investigate the combined effects of sleep quality and PA on the possibility of MAFLD. First, we determined the interaction between sleep quality and PA with MAFLD. Next, in the stratified analysis, we applied restricted cubic splines for sleep quality to estimate the association of the joint effects based on total PA or MVPA categories. Second, the relationship between sleep quality (good sleep as reference group) and MAFLD was examined at various levels of total PA or MVPA. Third, the combined association of sleep quality and PA was subsequently investigated using adjusted logistic regression analysis. Participants were stratified into nine distinct groups based on their total volume of PA and quality of sleep, with the group exhibiting high PA levels and good sleep quality serving as the baseline reference group. Similarly, a joint analysis was conducted on six distinct groups delineated by categories of MVPA and sleep quality.

Subgroup analysis stratified by gender, age, presence of metabolic comorbidities and several sensitivity analyses, were carried out to further assess the robustness of our findings. The analyses were replicated in the subset with complete data on all covariates, with additional adjustments made for prevalent metabolic comorbidities. The analysis was further restricted to individuals not taking any medications that could potentially influence sleep behaviors. The E-value method was utilized to assess residual confounding in the observed association [28].

All data analyses were conducted using Stata version 16.0 (StataCorp LLC, College Station, Texas, USA) or R version 4.2.3 (The R Foundation for Statistical Computing, Vienna, Austria). Statistical significance was defined as a two-tailed *P*-value less than 0.05.

## Results

### Baseline characteristics

Baseline characteristics of the study subjects with different sleep quality were shown in Table 1. Among the 10089 participants included, the mean (SD) age was 47.0 (9.1) years old, 5207 (51.6%) were men, and 3854 (38.2%) were diagnosed with MAFLD. The majority of the study participants (60.7%) had intermediate sleep quality, and only 12.3% of them had a high volume of PA. Notably, almost 80% of the study participants failed to meet the MVPA targets recommended by WHO. In comparison to individuals experiencing poor sleep quality, those with good sleep quality were more likely to be women, exhibit a lower prevalence of prevalent metabolic disorders, and possess more favorable metabolic profiles and lifestyles.

**Table 1:** Basic characteristics of study population according to sleep quality.

	Overall	Sleep quality			*P*-trend
		Good	Intermediate	Poor	
*n*	10089	2632 (26.1)	6127 (60.7)	1330 (13.2)	
Male, n (%)	5 207 (51.6)	1 102 (41.9)	3 326 (54.3)	779 (58.6)	**<** **0.001**
Age (y)	47.0 ± 9.1	47.0 ± 9.3	46.9 ± 9.0	47.5 ± 8.6	0.238
Ethnic, *n* (%)					0.072
Han	8660 (85.8)	2232 (84.8)	5275 (86.1)	1153 (86.7)	
Others	1429 (14.2)	400 (15.2)	852 (13.9)	177 (13.3)	
BMI (kg/m^2^)	24.8 ± 3.3	24.3 ± 3.3	24.1 ± 3.4	25.2 ± 3.3	**<** **0.001**
BMI status, *n* (%)					**<** **0.001**
< 24.0 (kg/m^2^)	4302 (42.6)	1 264 (48.0)	2 540 (41.5)	498 (37.4)	
24.0 to 27.9 (kg/m^2^)	4075 (40.4)	1 017 (38.6)	2 502 (40.8)	556 (41.8)	
≥ 28.0 (kg/m^2^)	1 712 (17.0)	351 (13.3)	1 085 (17.7)	276 (20.8)	
Married, *n* (%) ^a^	9 428 (93.6)	2 466 (93.8)	5 722 (93.6)	1 240 (93.4)	0.597
Higher education, *n* (%) ^a^	9 419 (93.5)	2 436 (92.7)	5 757 (94.1)	1 226 (92.3)	0.749
Comorbidities					
Diabetes mellitus, *n* (%) ^a^	790 (7.8)	184 (7.0)	486 (7.9)	120 (9.0)	**0.022**
Hypertension, *n* (%) ^a^	3 050 (30.2)	708 (26.9)	1 913 (31.2)	429 (32.3)	**<** **0.001**
Metabolic syndrome, *n* (%) ^a^	2 973 (29.6)	618 (23.6)	1 877 (30.8)	478 (36.1)	**<** **0.001**
Lifestyles					
Drinking, *n* (%)	5 833 (57.8)	1 386 (52.7)	3 634 (59.3)	813 (61.1)	**<** **0.001**
Current smoking, *n* (%)	2 359 (23.4)	339 (15.2)	1 527 (24.9)	433 (32.6)	**<** **0.001**
Sedentary time (h/day)^a^	5.0 (3.0–7.0)	5.0 (3.0–7.0)	5.0 (3.0–7.0)	5.0 (3.0–7.0)	0.496
Diet diversity score	4.8 ± 1.3	5.0 ± 1.2	4.8 ± 1.2	4.7 ± 1.3	**<** **0.001**
Physical and clinical measurements					
Waist (cm)^a^	85.0 ± 10.8	82.6 ± 10.6	85.5 ± 10.8	87.3 ± 10.8	**<** **0.001**
SBP (mmHg)^a^	125.6 ± 17.4	125.0 ± 18.0	125.8 ± 17.2	126.0 ± 17.0	0.064
DBP (mmHg)^a^	79.6 ± 12.1	78.9 ± 11.9	79.8 ± 12.1	80.0 ± 12.4	**0.002**
Fasting glucose (mmol/L)^a^	5.3 ± 1.4	5.2 ± 1.3	5.3 ± 1.4	5.4 ± 1.6	**<** **0.001**
TG (mmol/L)^a^	1.3 (0.9–2.0)	1.2 (0.9–1.8)	1.4 (0.9–2.0)	1.5 (1.0–2.2)	**<** **0.001**
TC (mmol/L)^a^	4.8 ± 1.0	4.8 ± 1.0	4.8 ± 1.0	4.9 ± 1.0	0.815
HDL-c (mmol/L)^a^	1.3 ± 0.3	1.3 ± 0.3	1.3 ± 0.3	1.2 ± 0.3	**<** **0.001**
LDL-c (mmol/L)^a^	3.2 ± 0.8	3.2 ± 0.8	3.2 ± 0.8	3.3 ± 0.8	0.086
ALT (IU/L)^a^	19.2 (13.8–28.1)	18.0 (13.1–25.9)	19.7 (14.0–28.7)	20.2 (14.1–29.2)	**<** **0.001**
AST (IU/L)^a^	20.3 (17.0–24.6)	20.0 (16.9–24.4)	20.3 (17.2–24.6)	20.7 (17.1–25.0)	**0.013**
Total volume of PA, *n* (%)					**0.009**
High	1242 (12.3)	336 (12.8)	747 (12.2)	159 (12.0)	
Medium	6649 (65.9)	1782 (67.7)	4003 (65.3)	864 (65.0)	
Low	2198 (21.8)	514 (19.5)	1377 (22.5)	307 (23.1)	
Recommended MVPA, *n* (%)					0.062
Yes	2102 (20.8)	578 (22.0)	1264 (20.6)	260 (19.5)	
No	7987 (79.2)	2054 (78.0)	4863 (79.4)	1 070 (80.5)	

Data were shown as *n* (%), mean (SD), or median (interquartile), as appropriate. *P* for trend was calculated by linear regression model or Mantel-Haenszel chi-square test. Total volume of PA was categorized as low (< 600 MET mins/week), medium (600 to 3000 MET mins/week), and high (> 3000 MET mins/week). MVPA was dichotomized as meeting or not meeting current guidelines (MVPA < 150 or ≥ 150 min). Sleep quality was categorized as poor (0–2), intermediate (3–4) and good (5–6).

*ALT* alanine aminotransferase, *AST* aspartate aminotransferase, *BMI* body mass index, *DBP* diastolic blood pressure, *HDL-c* high-density lipoprotein cholesterol, *LDL-c* low-density lipoprotein cholesterol, *MAFLD* metabolic dysfunction–associated fatty liver disease, *MVPA* moderate-to-vigorous physical activity; *PA* physical activity, *SBP* systolic blood pressure, *TC* total cholesterol, *TG* triglyceride.

^a^Number of participants with missing value were as follows: married (*n* = 19), higher education (*n* = 10), diabetes mellitus (*n* = 24), hypertension (*n* = 2), metabolic syndrome (*n* = 53), sedentary time (*n* = 24), waist (*n* = 2), SBP (*n* = 2), DBP (*n* = 2), fasting glucose (*n* = 24), TG (*n* = 16), TC (*n* = 17), HDL-c (*n* = 40), LDL-c (*n* = 40), ALT (*n* = 83), AST (*n* = 86).

Bold values indicates statistically significant differences.

### Independent associations of sleep quality and physical activity with MAFLD

**Fig. 1:**
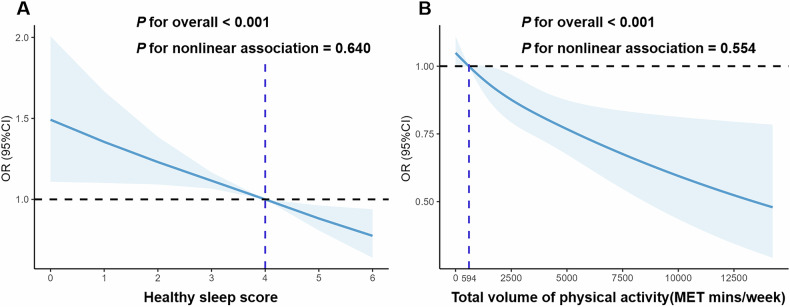
Independent association of sleep quality and physical activity with MAFLD. Risk of developing MAFLD according to (**A**) healthy sleep score and (**B**) total volume of physical activity (MET-mins/week). Restricted cubic splines were constructed with three knots located at the 5th, 50th, and 95th percentiles of each exposure. Multivariable models were adjusted for age, gender, higher education (yes or no), married (yes or no), current smoking, drinking, sedentary time, diet diversity score, BMI status and sleep quality/physical activity, as appropriate. Abbreviations: CI, confidence interval; OR, odds ratio.

Figure 1 and Table S3 showed the independent (and mutually adjusted) associations of sleep quality and PA with prevalent MAFLD. Sleep quality was associated with MAFLD prevalence in a linear manner (Fig. 1A). Compared to those with good sleep quality, the fully adjusted OR for the MAFLD (95% confidence interval [CI]) of those with intermediate and poor sleep quality were 1.25 (95% CI: 1.11–1.40) and 1.45 (95% CI: 1.23–1.70), respectively (*P* for trend < 0.001, Table S3).

A dose-dependent decrease in MAFLD prevalence was also found with increasing volume of total PA (Fig. 1B). Compared to individuals with a high volume of PA, those with all the other levels of PA had an incrementally higher possibility for developing MAFLD after full adjustment, with ORs of 1.24 (95% CI:1.07–1.44) for subjects with medium level of PA and 1.37 (95% CI:1.15–1.63) for those with low PA, respectively (Table S3**)**. Additionally, failure to meet the standard recommendation of MVPA proposed by WHO dramatically enhanced the possibility for having MAFLD by almost 40% (OR = 1.37, 95% CI:1.21–1.54, Table S3).

### Joint associations of sleep quality and physical activity with MAFLD

**Fig. 2:**
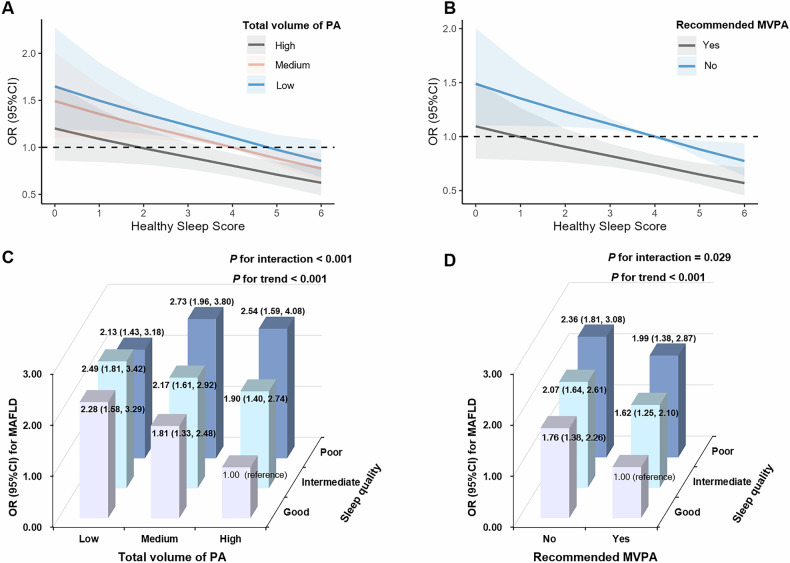
Joint associations of sleep quality and physical activity with MAFLD. Dose-dependent associations of sleep quality and (**A**) total volume of PA or (**B**) MVPA with MAFLD. Joint associations of sleep quality and (**C**) total volume of PA or (**D**) MVPA with MAFLD. Sleep quality was categorized as poor (0–2), intermediate (3-4) and good (5-6). Total volume of PA was categorized as low (< 600 MET mins/week), medium (600 to 3000 MET mins/week) and high (> 3000 MET mins/week). MVPA was dichotomized as meeting or not meeting current physical activity guidelines. *P* for interaction indicated the multiplicative interactions of sleep quality and physical activity. *P* for trend was calculated by linear regression model or Mantel-Haenszel chi-square test. Multivariable models were adjusted for age, gender, higher education (yes or no), married (yes or no), current smoking, drinking, sedentary time, diet diversity score and BMI status. CI confidence interval, MAFLD metabolic associated fatty liver disease, MVPA moderate-to-vigorous physical activity, OR odds ratio, PA physical activity.

Figure 2, Table 2 and Figure S3 demonstrated the associations of sleep quality with MAFLD prevalence stratified by categories of the total volume of PA and MVPA. Within each category of the total volume of PA, there was a notably decreasing trend of MAFLD prevalence with improving sleep quality (Fig. 2A). In subjects with high volume of PA, poor sleep quality led to additional 153% increases in the prevalence of MAFLD (OR = 2.53, 95% CI: 1.59–4.01). In the vast majority of individuals who had a medium level of PA, even a moderate reduction in sleep quality led to a 20% increases the prevalence for MAFLD (OR = 1.20, 95% CI: 1.04–1.38, Table 2). Similarly, regardless of the achievement of MVPA target, each additional increment in healthy sleep score led to a substantial decrease in MAFLD prevalence (Fig. 2B). Even in those who engaged in MVPA above the recommended target, having a poor sleep quality led to additional 101% increases in the prevalence for MAFLD (OR = 2.01, 95% CI: 1.40–2.90, Table 2). Notably, such a synergetic protection of sleep quality and PA against MAFLD was generally similar between men and women, as well as across various subgroups stratified by age, prevalence of hypertension, diabetes mellitus and metabolic syndrome (Fig. S3).

**Table 2:** Associations of sleep quality with MAFLD stratified by total volume of physical activity and moderate-to-vigorous physical activity.

Physical activity	Sleep quality	N	Cases (%)	Model 1	Model 2	Model 3
*Total volume of PA*						
High	Good	336	79 (23.5)	1.00 (reference)	1.00 (reference)	1.00 (reference)
	Intermediate	747	292 (39.1)	**1.92 (1.42, 2.59)**	**1.90 (1.42, 2.57)**	**2.00 (1.44, 2.78)**
	Poor	159	72 (45.3)	**2.57 (1.70, 3.88)**	**2.48 (1.64, 3.76)**	**2.53 (1.59, 4.01)**
*P*-trend				**<** **0.001**	**<** **0.001**	**<** **0.001**
Medium	Good	1782	572 (32.1)	1.00 (reference)	1.00 (reference)	1.00 (reference)
	Intermediate	4003	1605 (40.1)	**1.27 (1.12, 1.44)**	**1.26 (1.11, 1.42)**	**1.20 (1.04, 1.38)**
	Poor	864	406 (47.0)	**1.59 (1.34, 1.90)**	**1.56 (1.30, 1.86)**	**1.52 (1.25, 1.86)**
*P*-trend				**<** **0.001**	**<** **0.001**	**<** **0.001**
Low	Good	514	162 (31.5)	1.00 (reference)	1.00 (reference)	1.00 (reference)
	Intermediate	1377	539 (39.1)	1.15 (0.91, 1.46)	1.12 (0.89, 1.42)	1.05 (0.81, 1.36)
	Poor	307	127 (41.4)	1.14 (0.83, 1.57)	1.08 (0.78, 1.48)	0.86 (0.60, 1.24)
*P*-trend				0.480	0.616	0.465
Recommended MVPA						
Yes	Good	578	136 (23.5)	1.00 (reference)	1.00 (reference)	1.00 (reference)
	Intermediate	1264	439 (34.7)	**1.56 (1.23, 1.96)**	**1.53 (1.21, 1.94)**	**1.64 (1.27, 2.12)**
	Poor	260	105 (40.4)	**2.04 (1.47, 2.82)**	**1.94 (1.40, 2.70)**	**2.01 (1.40, 2.90)**
*P*-trend				**<** **0.001**	**<** **0.001**	**<** **0.001**
No	Good	2054	677 (33.0)	1.00 (reference)	1.00 (reference)	1.00 (reference)
	Intermediate	4863	1997 (41.1)	**1.25 (1.12, 1.41)**	**1.24 (1.10, 1.39)**	**1.16 (1.02, 1.33)**
	Poor	1070	500 (46.7)	**1.48 (1.26, 1.73)**	**1.43 (1.22, 1.68)**	**1.33 (1.11, 1.60)**
*P*-trend				**<** **0.001**	**<** **0.001**	**0.006**

Multivariate-adjusted logistic regression was used in this analysis. *P* for trend was calculated by Mantel-Haenszel chi-square test. Total volume of PA was categorized as low (< 600 MET mins/week), medium (600 to 3000 MET mins/week) and high (>3000 MET mins/week). MVPA was dichotomized as meeting or not meeting WHO guideline (MVPA < 150 or ≥150 min). Sleep quality was categorized as poor (0–2), intermediate (3–4) and good (5–6). Model 1: adjusted for age and gender; Model 2: Model 1 plus higher education (yes or no), married (yes or no), current smoking, drinking, sedentary time and diet diversity score; Model 3: Model 2 plus BMI status. *MVPA* moderate-to-vigorous physical activity, *PA* physical activity.

Bold values indicates statistically significant differences.

Regarding the joint associations of sleep quality and PA with MAFLD prevalence, the lowest possibility of MAFLD was observed in the group characterized by a high volume of PA and good sleep quality (Fig. 2C). The combination of a medium volume of PA with poor sleep quality was linked to the highest likelihood of developing MAFLD (OR = 2.73, 95% CI: 1.96–3.80), followed by the combination of high volume of PA with poor sleep quality (OR = 2.54, 95% CI: 1.59–4.08), and low volume of PA with intermediate sleep quality (OR = 2.49, 95% CI: 1.81–3.42). Likewise, when grouped by MVPA threshold, the ORs for MAFLD associated with poor and intermediate sleep quality among individuals not meeting the MVPA recommended target substantially declined by 37 and 45%, respectively (Fig. 2D). Moreover, we found significant multiplicative interactions of sleep quality, total volume of PA and MVPA with the possibility of having MAFLD (Fig. 2C, D).

### Sensitivity analyses

The results of all sensitivity analyses were consistent, for example by restricting the analysis to subjects with no missing data (Table S4), individuals with no history of mediations which may affect sleep behaviors (Table S5), and further adjustment for prevalent metabolic disorders (Table S6). Additionally, considering that most observed relationships between lifestyle factors and MAFLD rarely exceed a relative OR of 3.00, the E value analysis suggested that no residual confounding factors remained in our study (Table S7).

## Discussion

In the current societal landscape, there is a notable prevalence of sleep disorders and sedentary lifestyles, which contribute to the rising incidence of metabolic disorders. In this cross-sectional study of adults in Western China, a significant interaction between PA and sleep quality was observed in relation to the prevalence of MAFLD. The highest prevalence of MAFLD was identified in individuals with poor sleep quality and low to moderate levels of PA. In contrast, good sleep provided an additional reduction in the possibility of having MAFLD among participants with high volume of PA. Significantly, our research findings indicated that engaging in moderate to MVPA at levels exceeding the threshold recommended by the WHO did not effectively alleviate the negative correlation between poor sleep quality and MAFLD.

With dramatic changes in lifestyles over recent decades, MAFLD has emerged as the predominant liver disorder in China, posing a substantial burden to the society [29]. Concordant with the latest meta-analysis that northwest China witnessed a heavy disease burden of MAFLD [30], the prevalence of MAFLD was found to be 38.2% in our study, slightly higher than the national average prevalence of 29.2% [30]. Additionally, this study found that the prevalence of MAFLD in urban areas of Xinjiang was higher than the prevalence of 16.6% observed in the rural Uighur population in Kashgar, with the lower prevalence in Guo et al.‘s study attributed to a younger population, higher physical activity levels in rural areas, and ethnic differences [31]. Similar to our observation, a prevalence of 35.6% for MAFLD in western regions had also been found in a previous study conducted in health care centers across China [2]. Poor diet and insufficient PA were considered as the main drivers of MAFLD [6, 32, 33]. However, it was noticeable that the vast majority of the study population had a moderate diet diversity, whereas, 79.2% of them failed to achieve the MVPA target and only 12.3% of the participants had a high volume of PA (Table 1), considerably lower than the average level of PA previously reported in China [34]. In line with previous observations in the USA [35], UK [36] and Japan [37], a graded protective effect of PA was also observed in our study and engagement in MVPA above the recommended target by WHO resulted in nearly 40% decline in the prevalence of MAFLD (Table S3). Though different types of PA may affect metabolic disorders in different ways, both aerobic exercise and resistance exercise have been demonstrated to be able to reduce liver fat, improve metabolic parameters, and enhance cardiorespiratory fitness [38, 39]. Moreover, consistent with previous observations that poor sleep served as an emerging contributor to multiple metabolic disorders [13, 40, 41], less than 30% of the participants in our study reported a good sleep (Table 1). Sleep is an intricate and meticulously regulated physiological function that plays a pivotal role in sustaining human health and overall well-being [42]. Consistently, our study indicated that as sleep quality deteriorated, there was a dose-dependent escalation in the prevalence of MAFLD. Moreover, even a moderate decline in sleep quality resulted in 25% higher prevalence for MAFLD after adjustment for PA and diet (Table S3), largely consistent with the observation in Southern China [13] and Korea [43]. Researchers have proposed several explanations for the substantial correlation between poor sleep quality and MAFLD. For instance, a recent study has indicated that hepatic metabolism is highly dynamic and influenced by circadian rhythms [44, 45]. In addition, as a site of lipid synthesis in hepatocytes, various regulatory proteins were found to be under circadian control in endoplasmic reticulum [46]. Collectively, our findings suggest that such a high burden of MAFLD may partially due to a considerably lower volume of PA and poor sleep quality in this population.

Although a recent study has indicated a synergistic effect of insufficient exercise and poor sleep quality on overall and cardiovascular mortality [47, 48]. To our best knowledge, this is the first study to demonstrate a joint association of PA and sleep with MAFLD prevalence. Compared with those with the high PA-good sleep quality combination, participants with no MVPA-poor sleep quality combination had substantially higher prevalence of MAFLD. Notably, the detrimental effect of physical inactivity could potentially be reversed or largely diminished among participants with good sleep quality. Given the fact that the volume of PA was extremely low in this population, our findings highlight an urgent need to target sleep quality to further improve the effectiveness of MAFLD prevention among individuals with low PA. On the other hand, it should be noted that engaging in PA at or above the lower threshold recommended by WHO (600 MET-minutes/week) or achieving the recommended target for MVPA seemed not sufficient enough to attenuate the detrimental effects of poor sleep on MAFLD. Among participants who met the MVPA target proposed by WHO, suffering from intermediate or poor sleep led to an additional 62 and 99% increased prevalence for developing MAFLD (Fig. 2D). Even a high volume of PA could not offset the adverse effects of intermediate or poor sleep (Fig. 2C). Furthermore, the combined impact of PA and sleep quality on MAFLD was largely similar in all subgroups, irrespective of age, gender and the presence of comorbidities. Further clinical trials are needed to determine if the observed associations between sleep and PA are causal, and additional mechanistic studies are necessary to identify the biological mediators. Despite this need for further research, several potential explanations for the combined effects of sleep and PA have been suggested. For instance, PA had the potential to regulate circadian rhythm and ameliorate depressed mood [49, 50], which in turn improved sleep quality and metabolic health. On the other hand, sufficient sleep may mitigate physical inactivity by alleviating daytime fatigue and sleepiness [49, 50], which were also key contributors to metabolic dysfunctions. In conclusion, our research findings underscore the potential synergistic effects of interventions targeting both PA and sleep quality in adults, which may be more effective in preventing MAFLD than interventions focusing solely on either behavior. Maintaining PA is important but not sufficient for the management of MAFLD. Therefore, interventions targeting both engagement in MVPA and improvement in sleep quality should be implemented to further advance the prevention and treatment of MAFLD.

This study’s robustness is underscored by its substantial sample size and the diverse representation of participants from residential communities, enabling the exploration of relationships among stratified subgroups in a real-world context and potentially bolstering the applicability of our results. Nevertheless, it is important to acknowledge the limitations of this study. The cross-sectional design restricts the ability to establish causal relationships. Despite this limitation, our findings remained robust in various sensitivity analyses and were further supported by controlling for potential confounding variables. However, future prospective studies in diverse populations are still necessary. Secondly, our study employed self-reported data on sleep behaviors and PA, which may be subject to misclassification and recall bias. It is important to note that non-differential misclassification could potentially weaken the study results and underestimate the significance of the observed relationships. Thirdly, although different types of PA have different effects on the prevalence of fatty liver disease [19, 51], the aim of the study was to investigate the impact of total PA and meeting the recommended MVPA on the prevalence of MAFLD. Additionally, calculating total PA and meeting the recommended MVPA is a common practice in large cohort studies. Ultimately, hepatic steatosis was diagnosed via ultrasound imaging, without the presence of histological confirmation. Nevertheless, invasive examinations are deemed unsuitable for population-based epidemiological studies [52]. Currently, ultrasound remains the most commonly utilized diagnostic tool for assessing fatty liver in population-based studies [53].

Poor sleep was linked to a higher odds ratio of MAFLD, and these associations were notably intensified in participants with insufficient PA. The current lower threshold recommended by the PA guidelines is insufficient to mitigate the harmful association between poor sleep and MAFLD. Our research findings call for public awareness regarding the adverse effects of poor sleep on metabolism and support the inclusion of sleep in the current lifestyle intervention strategies for MAFLD.

### Supplementary information

Supplementary Materials

## Data Availability

The data support the findings of this study are available from the corresponding author, Prof. Yin-Ning Yang, upon reasonable request.
