# Supplementary material for: Joint association of sleep quality and physical activity with metabolic dysfunction-associated fatty liver disease: a population-based cross-sectional study in Western China
Source: Nutr Diabetes. 2024 Jul 22;14:54. doi: 10.1038/s41387-024-00312-3 (PMC11263340; doi:10.1038/s41387-024-00312-3)
Supplement: Supplementary file 1 — Supplementary Materials [file 41387_2024_312_MOESM1_ESM.pdf]

Supplementary Materials

Joint Association of Sleep Quality and Physical Activity with Metabolic Dysfunction-Associated Fatty Liver Disease: a population-based cross-sectional study in Western China

Ying Wang, MD[1,2,7], Qian Zhao, PhD[1,2,7], Jialu Yang, PhD[3], Yushan Wang, MD[4], Lei Deng, MBBS[5], Hamulati Xieyire, Mphil[1,2], Tuerxun Gulijiehere, Mphil[1,2], Mutalifu Munire, Mphil[1,2], Fen Liu, PhD[1,2], Xiaomei Li, PhD[1,2], Min Xia, PhD[3,8], Yan Liu, PhD[3,8], Yining Yang, PhD[1,2,6,8]

[1]State Key Laboratory of Pathogenesis, Prevention and Treatment of High Incidence Diseases in Central Asia, Department of Cardiology, First Affiliated Hospital of Xinjiang Medical University, Urumqi, China.

[2]Xinjiang Key Laboratory of Cardiovascular Disease Research, Clinical Medical Research Institute of Xinjiang Medical University, Urumqi, China.

[3]Guangdong Provincial Key Laboratory of Food, Nutrition and Health, and Department of Nutrition, School of Public Health, Sun Yat-sen University, Guangzhou, P.R. China.

[4]Center of Health Management, The First Affiliated Hospital of Xinjiang Medical University, Urumqi, China.

[5]Baoshihua Korla Hospital, Korla, China.

[6]Department of Cardiology, People's Hospital of Xinjiang Uygur Autonomous Region, Urumqi, China.

[7]These authors contributed equally: Ying Wang and Qian Zhao.

[8]These authors are co-corresponding authors: Yining Yang, Yan Liu and Min Xia.

Table of contents **Pages**

Supplementary Methods 3

Table S1 7

Table S2 9

Table S3 11

Table S4 13

Table S5 15

Table S6 18

Table S7 21

Figure S1 23

Figure S2 24

Figure S3 28

Supplementary References 30

Supplementary Methods

Population-based Cohort study of Chronic Diseases in Xinjiang

Population-based Cohort study of Chronic Diseases in Xinjiang (PCCDX) was a community-based prospective cohort conducted in 2 cities across Xinjiang Uygur Autonomous Region, using a two-stage stratified sampling method. Urumqi and Korla were selected as our investigation points to the represent the northern and southern parts of Xinjiang, respectively. Potentially eligible participants were identified through the household registration system and health records at primary clinics in each community. Invitations by phone calls or posters and flyers in the community were performed by trained staffs.

Detailed inclusion criteria for PCCDX were as follows: (1) aged between 30 to 74 years; (2) permanent residents who have lived in the place of investigation for more than half a year before the survey; (3) no severe disability or mental illness which may render them unable to complete the face-to-face survey; (4) voluntary to participate in the study and provided written informed consent.

Definition and assessment of MAFLD

The diagnosis of MAFLD was based on radiologically diagnosed fatty liver and the presence of any of the following three criteria: overweight/obesity (BMI ≥23 kg/m^2), presence of diabetes, or evidence of metabolic dysfunction (1). Metabolic dysfunction was defined as the presence of at least two of the following conditions: (1) waist circumference ≥90 cm in males and ≥80 cm in females, (2) blood pressure ≥130/85 mmHg or receiving antihypertensive medication, (3) fasting plasma triglycerides ≥1.70 mmol/L or receiving specific medication treatment, (4)

plasma high-density lipoprotein cholesterol <1.0 mmol/L in males and <1.3 mmol/L in females or receiving specific medication treatment, (5) prediabetes defined as fasting blood glucose of 5.6-6.9 mmol/L, 2-hour post-load glucose of 7.8-11.0 mmol/L, or hemoglobin Alc (HbA1c) of 5.7% to 6.4% (1).

Abdominal ultrasound was performed using a 3.5-MHz transducer (GE, E9) by experienced sonographers who were unaware of study design and blinded to laboratory values. Images were captured in a standard fashion with the subject in the supine position and with right arm raised above the head (2). An ultrasonographic diagnosis of fatty liver was defined as the presence of a diffuse increase of fine echoes in the liver parenchyma compared with the kidney or spleen parenchyma (3).

Definition and assessment of other covariates

Demographic characteristics, clinical history, lifestyles including drinking, smoking, self-reported dietary and physical activity were collected through structed questionnaires. Higher education was defined as high school and above, current drinking was defined as drinking at least once per week in the past year (4), and current smokers were defined as smoking at least one cigarette per day or 7 cigarettes per weak for more than half a year (5). The dietary diversity score was to assess the quality of diet (6). Self-reported dietary intake was collected based on frequency of consumption of 9 major food groups, including vegetables, whole grains, nuts, seafood, milk, eggs, fruits, red meat and beverages. Briefly, if the participants were classified as at low-risk for each dietary factor (daily vegetable intake, daily whole grains intake, weekly nuts intake ≥5 days; weekly seafood intake ≥1 day; weekly milk intake ≥3 days; weekly eggs

intake ≥3 days; 1≤ weekly fruits intake <5 days; weekly red meat intake <3 days; monthly beverages intake ≤3 days), he or she would receive a score of 1, otherwise receiving 0. Therefore, the diet diversity score ranged from 0 to 9, with a higher score indicating a more diversified diet (6).

Physical examinations were carried by trained personnel. Blood pressure was measured on the right upper arm in the sitting position after at least 10-15 minutes of rest using a validated digital automatic analyzer (Omron HEM-7136). Venous blood was taken after overnight fasting for 10-12 hours and subjected to clinical and laboratory analysis at central laboratory. Overweight was defined as BMI 24.0-27.9 kg/m^2 and obesity was defined as BMI ≥28 kg/m^2 for Chinese (7). Diabetes mellitus was defined as fasting glucose ≥7.0 mmol/L, self- reported diabetes mellitus previously diagnosed by physician or current usage of antidiabetic medications (8). Hypertension was defined as systolic blood pressure ≥140 mmHg, diastolic blood pressure ≥90 mmHg, self-reported hypertension previously diagnosed by physician or current use of antihypertensive medications (9). Metabolic syndrome was defined as central obesity (waist ≥90 cm in males and ≥80cm in females) plus any 2 of the following 4 conditions: (1) elevated TG: serum TG ≥1.7 mmol/L or specific treatment; (2) reduced HDL-c: HDL-c <1.03 mmol/L in males, <1.29 mmol/L in females or specific treatment; (3) elevated blood pressure: systolic blood pressure ≥130 mmHg, diastolic blood pressure ≥85 mmHg or treatment of previously diagnosed hypertension; (4) raised fasting glucose: fasting glucose ≥5.6 mmol/L or previously diagnosed type 2 diabetes according to International Diabetes Federation(2005) (10).

Minimally sufficient adjustment set

Covariates were collected at the same time when participants underwent abdominal ultrasound. Among the variables available in the surveys, we selected candidate covariates to adjust for the potential confounders based on existing literatures (1, 11) and empirical associations. The selected candidate covariates were age, gender, higher education (yes or no), married (yes or no), smoking (yes or no), drinking (yes or no), sedentary time, diet diversity score, BMI status, diabetes mellitus (yes or no), hypertension (yes or no), and metabolic syndrome (yes or no). The directed acyclic graph (**Figure S2**) based on previous evidence was used to choose the minimally sufficient adjustment set (MSAS) (12). According to the directed acyclic graph, only age, gender, higher education (yes or no), married (yes or no), smoking (yes or no), drinking (yes or no), sedentary time, diet diversity score, and BMI status were confounders and thus included in the MSAS; all the other candidate covariates were likely to be mediators between MAFLD and behavior changes (**Figure S2**) (13).

Table S1. Baseline characteristics of participants included or excluded from analyses due to missing information

	Included participants	Excluded participants	*P*-value
n	10 089	2 206	
Male, n (%)	5 207 (51.6)	1 201 (54.4)	0.016
Age (y)	47.0 ± 9.1	46.2 ± 9.5	<0.001
BMI (kg/m^2) [a]	24.8 ± 3.3	25.6 ± 4.5	<0.001
Married, n (%) [a]	9 428 (93.6)	2 025 (92.3)	0.023
Higher education, n (%) [a]	9 419 (93.5)	2 073 (94.4)	0.100
Total volume of PA, n (%)			<0.001
High	1 242 (12.3)	245 (11.1)	
Medium	6 649 (65.9)	1 338 (60.7)	
Low	2 198 (21.8)	623 (28.2)	
Recommended MVPA, n (%)			0.006

Yes	2 102 (20.8)	402 (18.2)
No	7 987 (79.2)	1 804 (81.8)

Data were shown as n (%), mean (SD), or median (interquartile), as appropriate. Differences between included participants and excluded participants were examined by t test or Mann-Whitney U test for continuous variables and chi-square test for categorical variables. Total volume of PA was categorized as low (<600 MET mins/week), medium (600 to 3 000 MET mins/week), and high (>3 000 MET mins/week). MVPA was dichotomized as meeting or not meeting current guidelines (MVPA <150 or ≥150 min).

Abbreviations: BMI, body mass index; MVPA, moderate-to-vigorous physical activity; PA, physical activity.

[a] Number of participants from included/ excluded participants with missing value were as follows: BMI (267 in excluded participants), married (19/12), higher education (10/10).

Table S2. The numbers (percentages) of included participants with missing covariates

**Covariates**	**N**	**%**
Any missing covariate	444	0.18
Married	19	0.19
Higher education	10	0.10
Diabetes mellitus	24	0.24
Hypertension	2	0.02
Metabolic syndrome	53	0.53
Sedentary time	24	0.24
Waist	2	0.02
SBP	2	0.02
DBP	2	0.02
Fasting glucose	24	0.24

TG	16	0.16
TC	17	0.17
HDL-c	40	0.40
LDL-c	40	0.40
ALT	83	0.82
AST	86	0.85

Abbreviations: ALT, alanine aminotransferase; AST, aspartate aminotransferase; DBP, diastolic blood pressure; HDL-c, high-density lipoprotein cholesterol; LDL-c, low-density lipoprotein cholesterol; SBP, systolic blood pressure; TC, total cholesterol; TG, triglyceride.

Table S3. Independent association of sleep and physical activity with MAFLD

Exposure	N	Cases (%)	Model 1	Model 2	Model 3
**Sleep quality**					
Good	2 632	813 (30.9)	1.00 (reference)	1.00 (reference)	1.00 (reference)
Intermediate	6 127	2 436 (39.8)	**1.31 (1.18, 1.45)**	**1.29 (1.17, 1.44)**	**1.25 (1.11, 1.40)**
Poor	1 330	605 (45.5)	**1.58 (1.37, 1.82)**	**1.53 (1.32, 1.77)**	**1.45 (1.23, 1.70)**
***P*-trend**			**<0.001**	**<0.001**	**<0.001**
**Physical activity**					
High	1 242	443 (35.7)	1.00 (reference)	1.00 (reference)	1.00 (reference)
Medium	6 649	2 583 (38.8)	**1.31 (1.14, 1.49)**	**1.30 (1.13, 1.48)**	**1.24 (1.07, 1.44)**
Low	2 198	828 (37.7)	**1.39 (1.19, 1.62)**	**1.36 (1.16, 1.58)**	**1.37 (1.15, 1.63)**
***P*-trend**			**<0.001**	**<0.001**	**0.002**
**Recommended MVPA**					

Yes	2 102	680 (32.4)	1.00 (reference)	1.00 (reference)	1.00 (reference)
No	7 987	3 174 (39.7)	**1.54 (1.39, 1.72)**	**1.52 (1.36, 1.69)**	**1.37 (1.21, 1.54)**

Multivariate-adjusted logistic regression was used in this analysis. Two lifestyles (sleep quality and physical activity) were included simultaneously in the same model. *P* for trend was calculated by Mantel-Haenszel chi-square test. Total volume of PA was categorized as low (<600 MET mins/week), medium (600 to 3 000 MET mins/week), and high (>3 000 MET mins/week), with the high volume of PA as the reference group. MVPA was dichotomized as meeting or not meeting current physical activity guidelines (MVPA <150 or ≥150 min), with achievement of recommended MVPA as the reference group. Sleep quality was categorized as poor (0-2), intermediate (3-4) and good (5-6), with healthy sleep quality as the reference group. Model 1: adjusted for age and gender; Model 2: Model 1 plus higher education (yes or no), married (yes or no), current smoking, drinking, sedentary time and diet diversity score; Model 3: Model 2 plus BMI status.

Table S4. Joint associations of sleep quality and physical activity for MAFLD with complete dataset

Physical activity	Sleep quality	N	Cases (%)	OR (95%CI)	*P*-interaction	*P*-trend
**Total volume of PA**					**<0.001**	**<0.001**
High	Good	335	79 (23.6)	1.00 (reference)		
	Intermediate	743	290 (39.0)	**1.96 (1.40, 2.74)**		
	Poor	159	72 (45.3)	**2.54 (1.58, 4.08)**		
Medium	Good	1 772	571 (32.2)	**1.82 (1.33, 2.49)**		
	Intermediate	3 992	1 603 (40.2)	**2.17 (1.61, 2.92)**		
	Poor	857	405 (47.3)	**2.74 (1.97, 3.82)**		
Low	Good	510	160 (31.4)	**2.29 (1.59, 3.30)**		
	Intermediate	1 365	534 (39.1)	**2.47 (1.79, 3.40)**		
	Poor	305	126 (41.3)	**2.14 (1.43, 3.20)**		
**Recommended MVPA**					**0.030**	**<0.001**

Yes	Good	575	136 (23.7)	1.00 (reference)
	Intermediate	1 260	438 (34.8)	**1.62 (1.25, 2.10)**
	Poor	259	105 (40.5)	**1.99 (1.38, 2.87)**
No	Good	2 042	674 (33.0)	**1.76 (1.37, 2.25)**
	Intermediate	4 840	1 989 (41.1)	**2.05 (1.63, 2.59)**
	Poor	1 062	498 (46.9)	**2.36 (1.81, 3.08)**

Multivariate-adjusted logistic regression was used in this analysis. *P* for interaction indicated the multiplicative interactions of sleep quality and physical activity. *P* for trend was calculated by Mantel-Haenszel chi-square test. Total volume of PA was categorized as low (<600 MET mins/week), medium (600 to 3 000 MET mins/week) and high (>3 000 MET mins/week). MVPA was dichotomized as meeting or not meeting WHO guideline (MVPA <150 or ≥150 min). Sleep quality was categorized as poor (0-2), intermediate (3-4) and good (5-6). Multivariable models were adjusted for age, gender, higher education (yes or no), married (yes or no), current smoking, drinking, sedentary time, diet diversity score and BMI status. Abbreviations: CI, confidence interval; MAFLD, metabolic associated fatty liver disease; MVPA, moderate-to-vigorous physical activity; OR, odds ratio; PA, physical activity.

Table S5. Joint associations of sleep quality and physical activity with MAFLD in subjects with no history of mediations which may affect sleep behaviors

Physical activity	Sleep quality	N	Cases (%)	OR (95%CI)	*P*-interaction	*P*-trend
**Total volume of PA**					**0.001**	**<0.001**
High	Good	331	79 (23.9)	1.00 (reference)		
	Intermediate	724	283 (39.1)	**1.92 (1.37, 2.69)**		
	Poor	144	67 (46.5)	**2.80 (1.72, 4.56)**		
Medium	Good	1 756	562 (32.0)	**1.78 (1.30, 2.43)**		
	Intermediate	3 871	1 566 (40.5)	**2.17 (1.60, 2.93)**		
	Poor	800	377 (47.1)	**2.64 (1.89, 3.70)**		
Low	Good	505	160 (31.7)	**2.27 (1.57, 3.28)**		
	Intermediate	1 343	532 (39.6)	**2.52 (1.82, 3.47)**		
	Poor	288	124 (43.1)	**2.30 (1.53, 3.45)**		

**Recommended MVPA**					**0.028**	**<0.001**
Yes	Good	568	133 (23.4)	1.00 (reference)		
	Intermediate	1 216	424 (34.9)	**1.61 (1.24, 2.10)**		
	Poor	238	99 (41.6)	**2.07 (1.42, 3.02)**		
No	Good	2 024	668 (33.0)	**1.75 (1.37, 2.25)**		
	Intermediate	4 722	1 957 (41.4)	**2.09 (1.66, 2.65)**		
	Poor	994	469 (47.2)	**2.39 (1.82, 3.13)**		

Multivariate-adjusted logistic regression was used in this analysis. *P* for interaction indicated the multiplicative interactions of sleep quality and physical activity. *P* for trend was calculated by Mantel-Haenszel chi-square test. Total volume of PA was categorized as low (<600 MET mins/week), medium (600 to 3 000 MET mins/week) and high (>3 000 MET mins/week). MVPA was dichotomized as meeting or not meeting WHO guideline (MVPA <150 or ≥150 min). Sleep quality was categorized as poor (0-2), intermediate (3-4) and good (5-6). Multivariable models were adjusted for age, gender, higher education (yes or no), married (yes or no), current smoking, drinking, sedentary time, diet diversity score

and BMI status. Abbreviations: CI, confidence interval; MAFLD, metabolic associated fatty liver disease; MVPA, moderate-to-vigorous physical activity; OR, odds ratio; PA, physical activity.

Table S6. Joint associations of sleep quality and physical activity with MAFLD in subjects with further adjustment for prevalent metabolic comorbidities

**Physical activity**	**Sleep quality**	**N**	**Cases (%)**	**OR (95%CI)**	***P*-interaction**	***P*-trend**
**Total volume of PA**					**0.001**	**<0.001**
High	Good	336	79 (23.5)	1.00 (reference)		
	Intermediate	747	292 (39.1)	**1.85 (1.31, 2.63)**		
	Poor	159	72 (45.3)	**2.24 (1.37, 3.67)**		
Medium	Good	1 782	572 (32.1)	**1.70 (1.23, 2.35)**		
	Intermediate	4 003	1 605 (40.1)	**1.95 (1.43, 2.65)**		
	Poor	864	406 (47.0)	**2.42 (1.72, 3.40)**		
Low	Good	514	162 (31.5)	**2.07 (1.42, 3.02)**		
	Intermediate	1 377	539 (39.1)	**2.22 (1.60, 3.09)**		
	Poor	307	127 (41.4)	**1.82 (1.20, 2.76)**		

**Recommended MVPA**					**0.041**	**<0.001**
Yes	Good	578	136 (23.5)	1.00 (reference)		
	Intermediate	1 264	439 (34.7)	**1.53 (1.17, 2.00)**		
	Poor	260	105 (40.4)	**1.84 (1.26, 2.70)**		
No	Good	2 054	677 (33.0)	**1.60 (1.24, 2.07)**		
	Intermediate	4 863	1 997 (41.1)	**1.81 (1.43, 2.30)**		
	Poor	1 070	500 (46.7)	**2.01 (1.53, 2.64)**		

Multivariate-adjusted logistic regression was used in this analysis. *P* for interaction indicated the multiplicative interactions of sleep quality and physical activity. *P* for trend was calculated by Mantel-Haenszel chi-square test. Total volume of PA was categorized as low (<600 MET mins/week), medium (600 to 3 000 MET mins/week) and high (>3 000 MET mins/week). MVPA was dichotomized as meeting or not meeting WHO guideline (MVPA <150 or ≥150 min). Sleep quality was categorized as poor (0-2), intermediate (3-4) and good (5-6). Multivariable models were adjusted for age, gender, higher education (yes or no), married (yes or no), current smoking, drinking, sedentary time, diet diversity score,

BMI status, diabetes mellitus, hypertension, and metabolic syndrome. Abbreviations: CI, confidence interval; MAFLD, metabolic associated fatty liver disease; MVPA, moderate-to-vigorous physical activity; OR, odds ratio; PA, physical activity.

Table S7. E-values to assess multivariable adjusted OR that an unmeasured confounder would need to have with both the exposure and outcome to fully explain the observed joint associations of sleep quality and physical activity with MAFLD

Physical activity	Sleep quality	OR (95%CI)	E-value for OR
**Total volume of PA**			
High	Good	1.00 (reference)	1.00 (reference)
	Intermediate	**1.96 (1.40, 2.74)**	**3.33**
	Poor	**2.54 (1.59, 4.08)**	**4.52**
Medium	Good	**1.81 (1.33, 2.48)**	**3.02**
	Intermediate	**2.17 (1.61, 2.92)**	**3.76**
	Poor	**2.73 (1.96, 3.80)**	**4.90**
Low	Good	**2.28 (1.58, 3.29)**	**3.99**
	Intermediate	**2.49 (1.81, 3.42)**	**4.42**
	Poor	**2.13 (1.43, 3.18)**	**3.68**

**Recommended MVPA**			
Yes	Good	1.00 (reference)	1.00 (reference)
	Intermediate	**1.62 (1.25, 2.10)**	**2.62**
	Poor	**1.99 (1.38, 2.87)**	**3.39**
No	Good	**1.76 (1.38, 2.26)**	**2.92**
	Intermediate	**2.07 (1.64, 2.61)**	**3.56**
	Poor	**2.36 (1.81, 3.08)**	**4.15**

Multivariate-adjusted logistic regression was used in this analysis. Total volume of PA was categorized as low (<600 MET mins/week), medium (600 to 3 000 MET mins/week) and high (>3 000 MET mins/week). MVPA was dichotomized as meeting or not meeting WHO guideline (MVPA <150 or ≥150 min). Sleep quality was categorized as poor (0-2), intermediate (3-4) and good (5-6). Multivariable models were adjusted for age, gender, higher education (yes or no), married (yes or no), current smoking, drinking, sedentary time, diet diversity score, and BMI status. Abbreviations: CI, confidence interval; MAFLD, metabolic associated fatty liver disease; MVPA, moderate-to-vigorous physical activity; OR, odds ratio; PA, physical activity.

Figure S1. Flow chart of study population

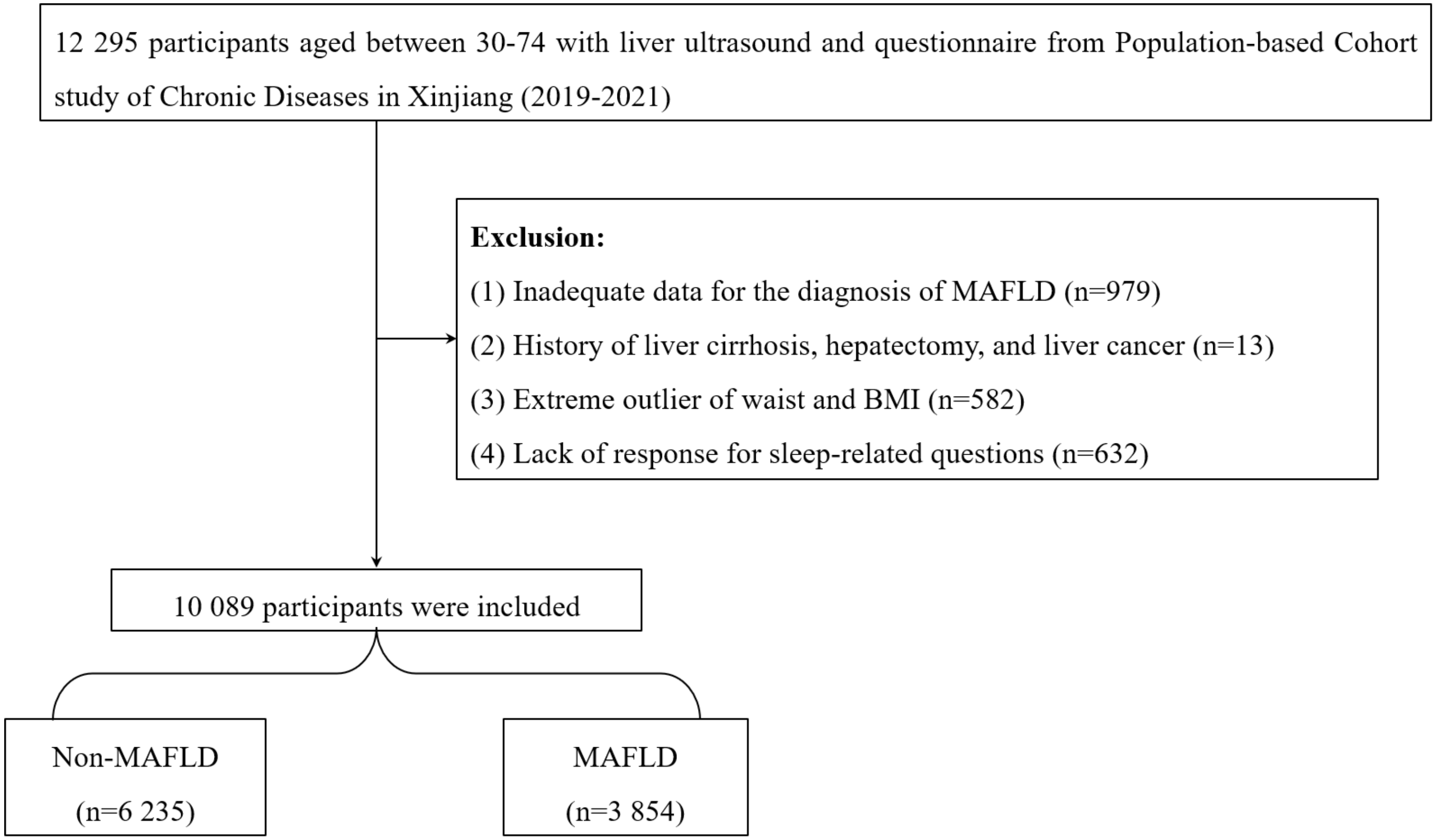

Abbreviations: MAFLD, metabolic-dysfunction associated fatty liver disease.

Figure S2. Directed acyclic graph showing potential causal pathways between variables of interest

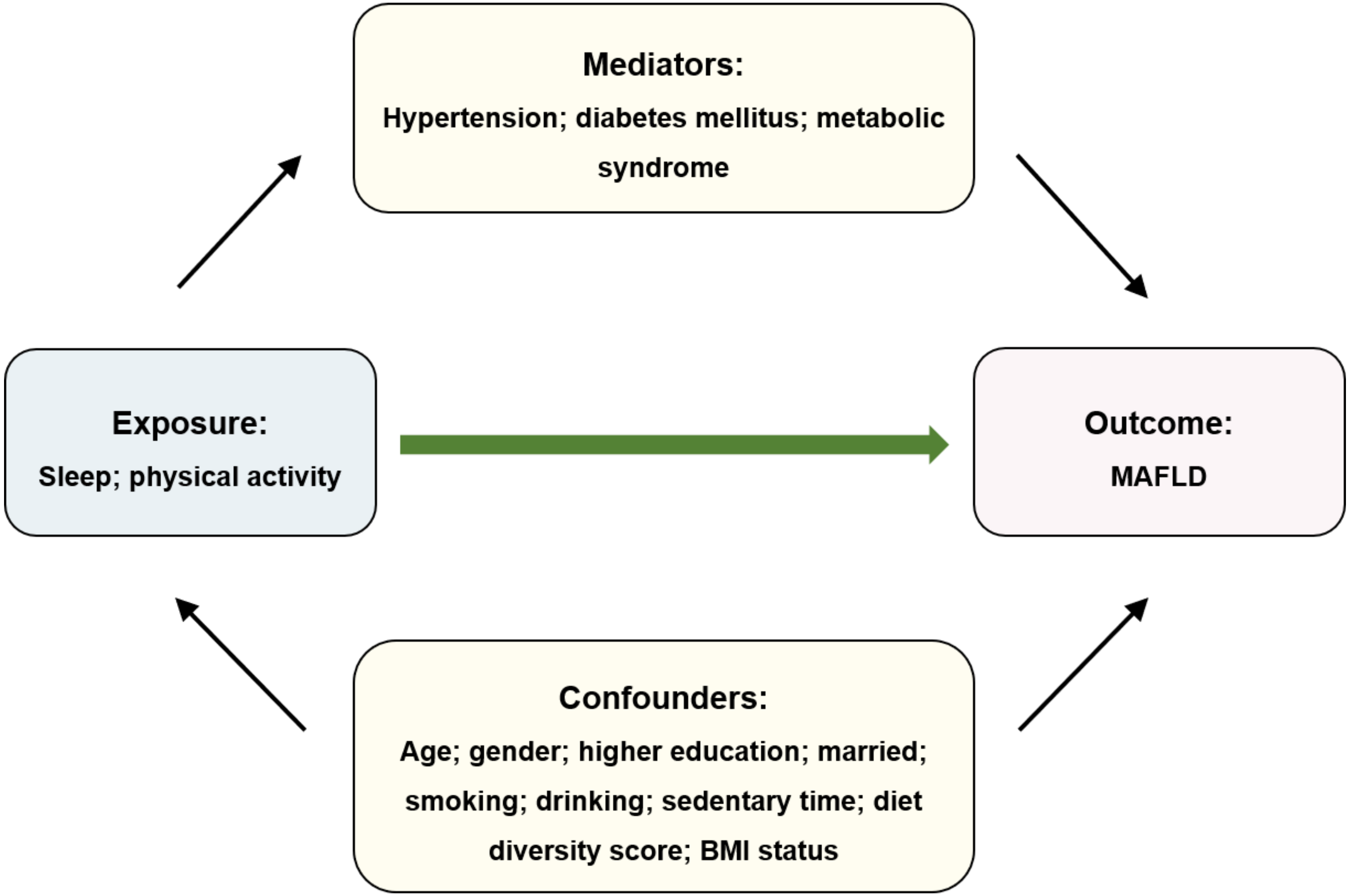

According to the directed acyclic graph (DAG), the main effect of interest is shown by the bolded green arrow. Regarding mediators (hypertension, diabetes mellitus and metabolic syndrome), since the exposure (sleep and physical activity) can affect mediators (hypertension, diabetes mellitus and metabolic syndrome) (14-19), and mediators (hypertension, diabetes mellitus and metabolic syndrome) in turn can affect the outcome (MAFLD) (20-22). The mediators should not be adjusted(13), therefore, based on the DAG, the minimally sufficient adjustment sets (MSAS) are age, gender, higher education, married, smoking, drinking, sedentary time, diet diversity score, and BMI status.

Main References:

13. Schisterman EF, Cole SR, Platt RW. Overadjustment bias and unnecessary adjustment in epidemiologic studies. Epidemiology (Cambridge, Mass). 2009;20(4):488-95.

Association between exposures (sleep and physical activity) and mediators (hypertension, diabetes mellitus, and metabolic syndrome):

14. Fernandez-Mendoza J, He F, Calhoun SL, Vgontzas AN, Liao D, Bixler EO. Association of Pediatric Obstructive Sleep Apnea With Elevated Blood Pressure and Orthostatic Hypertension in Adolescence. JAMA cardiology. 2021;6(10):1144-51.

15. Li Q, Li R, Zhang S, Zhang Y, He P, Zhang Z, et al. Occupational Physical Activity and New-Onset Hypertension: A Nationwide Cohort Study in China. Hypertension (Dallas, Tex : 1979). 2021;78(1):220-9.

16. Shan Z, Ma H, Xie M, Yan P, Guo Y, Bao W, et al. Sleep duration and risk of type 2 diabetes: a meta-analysis of prospective studies. Diabetes care. 2015;38(3):529-37.

17. Kyu HH, Bachman VF, Alexander LT, Mumford JE, Afshin A, Estep K, et al. Physical activity and risk of breast cancer, colon cancer, diabetes, ischemic heart disease, and ischemic stroke events: systematic review and dose-response meta-analysis for the Global Burden of Disease Study 2013. BMJ (Clinical research ed). 2016;354:i3857.

18. Xie J, Li Y, Zhang Y, Vgontzas AN, Basta M, Chen B, et al. Sleep duration and metabolic syndrome: An updated systematic review and meta-analysis. Sleep medicine reviews. 2021;59:101451.

19. Sagawa N, Rockette-Wagner B, Azuma K, Ueshima H, Hisamatsu T, Takamiya T, et al. Physical activity levels in American and Japanese men from the ERA-JUMP Study and associations with metabolic syndrome. Journal of sport and health science. 2020;9(2):170-8.

Association between mediators (hypertension, diabetes mellitus, and metabolic syndrome) and outcome (MAFLD):

20. Kasper P, Martin A, Lang S, Demir M, Steffen HM. Hypertension in NAFLD: An uncontrolled burden. Journal of hepatology. 2021;74(5):1258-60.

21. Vilar-Gomez E. NAFLD and liver-related events: does type 2 diabetes have a key role? The lancet Gastroenterology & hepatology. 2023.

22. Moore JB. Non-alcoholic fatty liver disease: the hepatic consequence of obesity and the metabolic syndrome. The Proceedings of the Nutrition Society. 2010;69(2):211-20.

Abbreviations: BMI, body mass index; MAFLD, metabolic dysfunction–associated fatty liver disease.

Figure S3. Joint associations of sleep quality and physical activity with MAFLD in defined subgroups

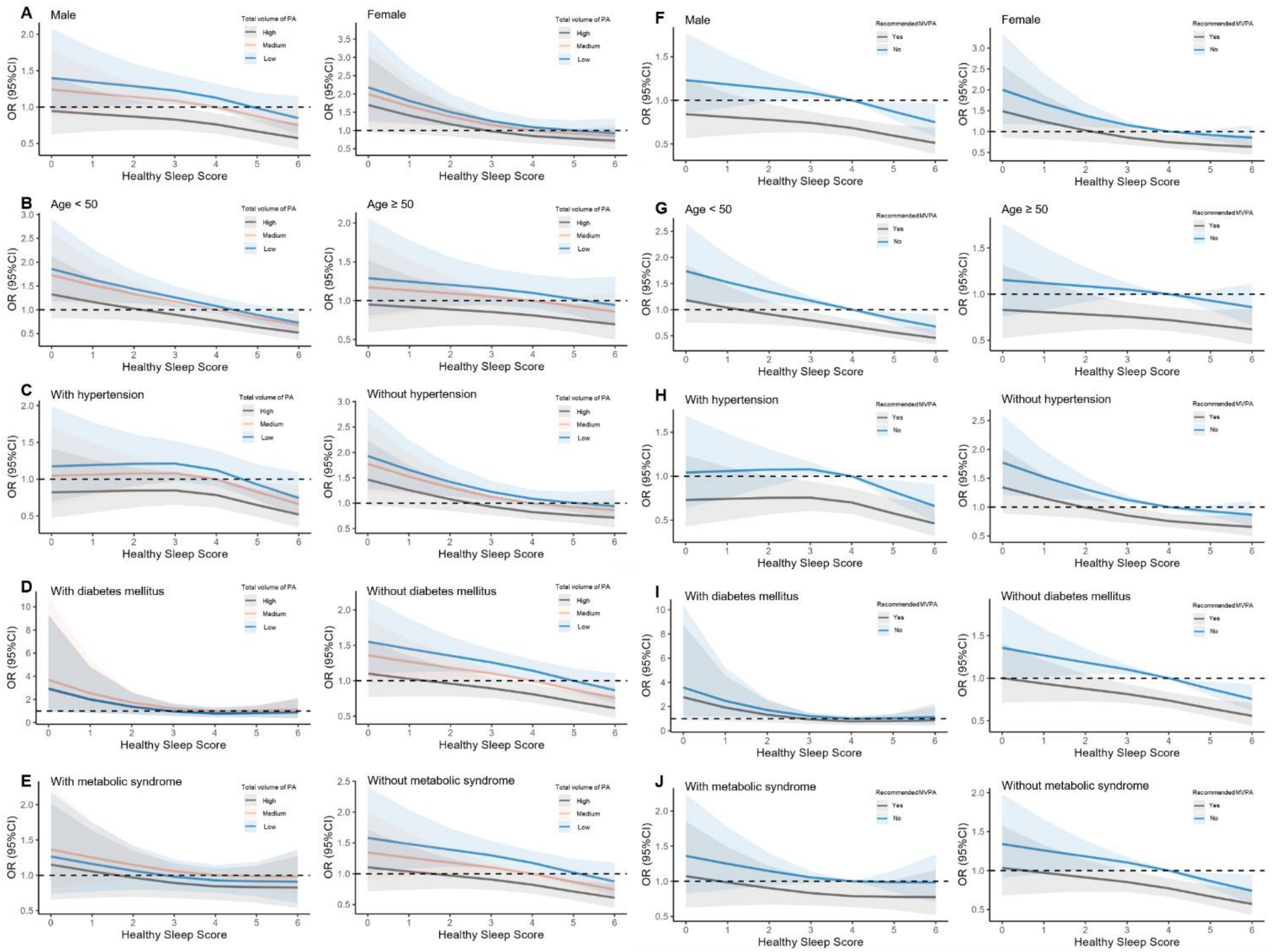

(A-E) Dose-dependent associations of sleep quality and total volume of PA with MAFLD in (A) male and female, (B) individuals younger or older than 50 years, (C) participants with or without hypertension, (D) subjects with or without diabetes, and (E) individuals with or without metabolic syndrome. (F-J) Dose-dependent associations of sleep quality and MVPA with MAFLD in (F) male and female, (G) individuals younger or older than 50 years, (H) participants with or without hypertension, (I) subjects with or without diabetes, and (J) individuals with or without metabolic syndrome. Restricted cubic splines were constructed with three knots located at the 5th, 50th, and 95th percentiles of each exposure. Total volume of PA was categorized as low (<600 MET mins/week), medium (600 to 3 000 MET mins/week) and high (>3 000 MET mins/week). Multivariable models were adjusted for age, gender, higher education (yes or no), married (yes or no), smoking, drinking, sedentary time, diet quality, BMI status and sleep quality/physical activity, as appropriate.

Supplementary References

1. Eslam M, Newsome PN, Sarin SK, Anstee QM, Targher G, Romero-Gomez M, et al. A new definition for metabolic dysfunction-associated fatty liver disease: An international expert consensus statement. Journal of hepatology. 2020;73(1):202-9.

2. Kim CW, Yun KE, Jung HS, Chang Y, Choi ES, Kwon MJ, et al. Sleep duration and quality in relation to non-alcoholic fatty liver disease in middle-aged workers and their spouses. Journal of hepatology. 2013;59(2):351-7.

3. Mathiesen UL, Franzén LE, Aselius H, Resjö M, Jacobsson L, Foberg U, et al. Increased liver echogenicity at ultrasound examination reflects degree of steatosis but not of fibrosis in asymptomatic patients with mild/moderate abnormalities of liver transaminases. Digestive and liver disease : official journal of the Italian Society of Gastroenterology and the Italian Association for the Study of the Liver. 2002;34(7):516-22.

4. Millwood IY, Walters RG, Mei XW, Guo Y, Yang L, Bian Z, et al. Conventional and genetic evidence on alcohol and vascular disease aetiology: a prospective study of 500 000 men and women in China. Lancet (London, England). 2019;393(10183):1831-42.

5. Yang G, Fan L, Tan J, Qi G, Zhang Y, Samet JM, et al. Smoking in China: findings of the 1996 National Prevalence Survey. Jama. 1999;282(13):1247-53.

6. Yin Z, Fei Z, Qiu C, Brasher MS, Kraus VB, Zhao W, et al. Dietary Diversity and Cognitive Function among Elderly People: A Population-Based Study. The journal of

nutrition, health & aging. 2017;21(10):1089-94.

7. Pan XF, Wang L, Pan A. Epidemiology and determinants of obesity in China. The lancet Diabetes & endocrinology. 2021;9(6):373-92.

8. Zhou L, Yu K, Yang L, Wang H, Xiao Y, Qiu G, et al. Sleep duration, midday napping, and sleep quality and incident stroke: The Dongfeng-Tongji cohort. Neurology. 2020;94(4):e345-e56.

9. Whelton PK, Carey RM, Aronow WS, Casey DE, Jr., Collins KJ, Dennison Himmelfarb C, et al. 2017 ACC/AHA/AAPA/ABC/ACPM/AGS/APhA/ASH/ASPC/NMA/PCNA Guideline for the Prevention, Detection, Evaluation, and Management of High Blood Pressure in Adults: A Report of the American College of Cardiology/American Heart Association Task Force on Clinical Practice Guidelines. Hypertension (Dallas, Tex : 1979). 2018;71(6):e13-e115.

10. Alberti KG, Zimmet P, Shaw J. Metabolic syndrome--a new world-wide definition. A Consensus Statement from the International Diabetes Federation. Diabetic medicine : a journal of the British Diabetic Association. 2006;23(5):469-80.

11. Yang J, Luo S, Li R, Ju J, Zhang Z, Shen J, et al. Sleep Factors in Relation to Metabolic Dysfunction-Associated Fatty Liver Disease in Middle-Aged and Elderly Chinese. The Journal of clinical endocrinology and metabolism. 2022;107(10):2874-82.

12. Tennant PWG, Murray EJ, Arnold KF, Berrie L, Fox MP, Gadd SC, et al. Use of directed acyclic graphs (DAGs) to identify confounders in applied health research:

review and recommendations. International journal of epidemiology. 2021;50(2):620-32.

13. Schisterman EF, Cole SR, Platt RW. Overadjustment bias and unnecessary adjustment in epidemiologic studies. Epidemiology (Cambridge, Mass). 2009;20(4):488-95.

14. Fernandez-Mendoza J, He F, Calhoun SL, Vgontzas AN, Liao D, Bixler EO. Association of Pediatric Obstructive Sleep Apnea With Elevated Blood Pressure and Orthostatic Hypertension in Adolescence. JAMA cardiology. 2021;6(10):1144-51.

15. Li Q, Li R, Zhang S, Zhang Y, He P, Zhang Z, et al. Occupational Physical Activity and New-Onset Hypertension: A Nationwide Cohort Study in China. Hypertension (Dallas, Tex : 1979). 2021;78(1):220-9.

16. Shan Z, Ma H, Xie M, Yan P, Guo Y, Bao W, et al. Sleep duration and risk of type 2 diabetes: a meta-analysis of prospective studies. Diabetes care. 2015;38(3):529-37.

17. Kyu HH, Bachman VF, Alexander LT, Mumford JE, Afshin A, Estep K, et al. Physical activity and risk of breast cancer, colon cancer, diabetes, ischemic heart disease, and ischemic stroke events: systematic review and dose-response meta-analysis for the Global Burden of Disease Study 2013. BMJ (Clinical research ed). 2016;354:i3857.

18. Xie J, Li Y, Zhang Y, Vgontzas AN, Basta M, Chen B, et al. Sleep duration and metabolic syndrome: An updated systematic review and meta-analysis. Sleep medicine reviews. 2021;59:101451.

19. Sagawa N, Rockette-Wagner B, Azuma K, Ueshima H, Hisamatsu T, Takamiya T,

et al. Physical activity levels in American and Japanese men from the ERA-JUMP Study and associations with metabolic syndrome. Journal of sport and health science. 2020;9(2):170-8.

20. Kasper P, Martin A, Lang S, Demir M, Steffen HM. Hypertension in NAFLD: An uncontrolled burden. Journal of hepatology. 2021;74(5):1258-60.

21. Vilar-Gomez E. NAFLD and liver-related events: does type 2 diabetes have a key role? The lancet Gastroenterology & hepatology. 2023.

22. Moore JB. Non-alcoholic fatty liver disease: the hepatic consequence of obesity and the metabolic syndrome. The Proceedings of the Nutrition Society. 2010;69(2):211-20.
